# Primary Meningococcal Polyarthritis in an Adult Woman

**DOI:** 10.1155/2015/563672

**Published:** 2015-03-08

**Authors:** José Celso Giordan Cavalcanti Sarinho, Marília Soares e Silva Arcadipane, Graziela Tavares Miola Menezes, Danilo Fernando Costa Duarte, Waldenise Cossermelli, Ivan Aprahamian

**Affiliations:** Department of Internal Medicine, Faculty of Medicine of Jundiaí, 13202-550 Jundiaí, SP, Brazil

## Abstract

Primary joint infection caused by the Gram-negative bacteria *Neisseria meningitidis* is rare. Normally, joint involvement comes secondary to meningitis or severe sepsis caused by this agent. When primary arthritis is seen, monoarthritis is the most common presentation. A meningococcal polyarthritis is described in less than 10 case reports according to current literature. This case report aims to briefly review this rare clinical event in an adult woman with no previous history of rheumatological disease. Early diagnosis of polyarthritis caused by meningococcal bacteria usually present a good prognosis when properly treated.

## 1. Introduction

Meningococcal arthritis without signs or symptoms of meningitis or meningococcemia is a rare manifestation of the disease in adults [1]. It is characterized by a variable joint involvement, generally with good prognosis when properly treated. Normally, monoarthritis of the knee is the most typical presentation [2]. It is often preceded by an infection of the upper airways as well as in meningitis or sepsis [3]. Mild or even asymptomatic infection by meningococcal bacteria is rare in adults, being more common among children [4]. We present a rare case of polyarthritis related to an infection by* Neisseria meningitidis* of the serogroup C in an adult woman.

## 2. Case Report

**Figure 1:**
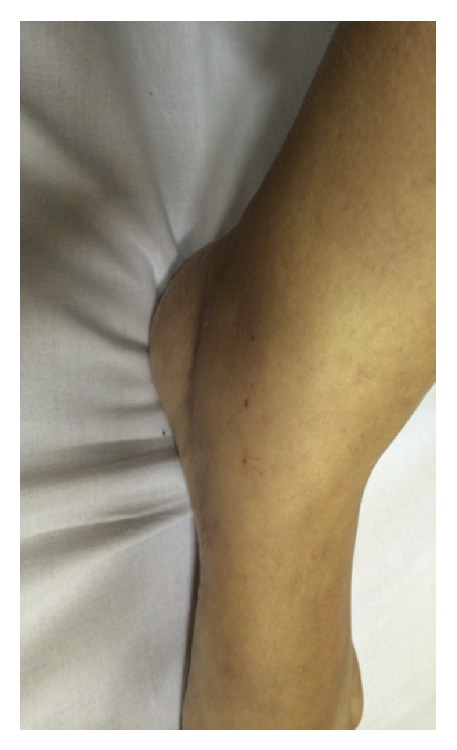
Arthritis of the left ankle with discrete petechiae.

**Figure 2:**
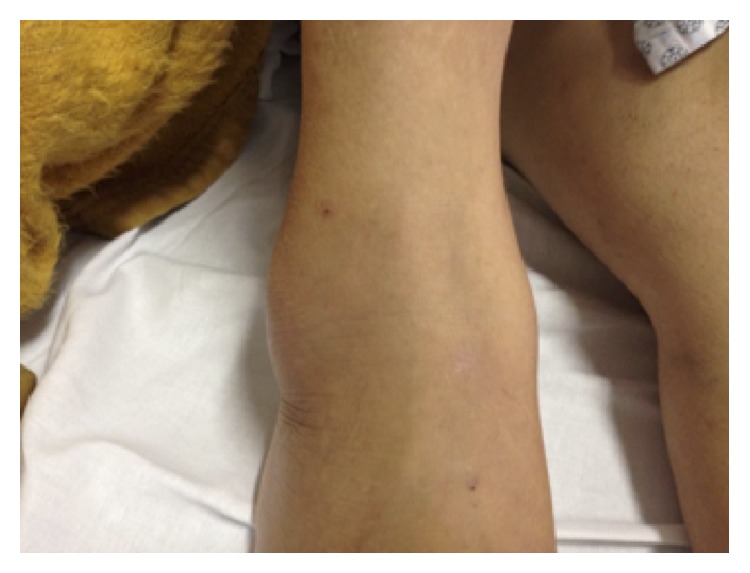
Arthritis of the right ankle.

A 53-year-old female patient was admitted to the emergency department with 4-day progressive polyarthritis. Previously, the patient presented with controlled systemic hypertension and recovery from a major depression a couple of years ago. She started with a throat pain 10 days before admission. An anti-inflammatory (sodium diclofenac) was prescribed by a general practitioner for three days with improvement of the pain. Four days after the sore throat she presented with edema, pain, and hyperemia at her right ankle progressively involving her left ankle, knees, wrists, and elbows, followed by prolonged morning stiffness (around 2 hours) and diffuse erythema in both legs. She also reported fever twice, since she started a sore throat, with 38 and 38,5 degrees Celsius. There was no headache or gastrointestinal or urinary symptoms. There was no sign of pulmonary involvement too. Her close contacts had been well. At the admission, there were no signs of sepsis or neurological alterations including meningeal signs at her physical exam. She presented without any temperature alterations, with no tachycardia and normal blood pressure, and she was breathing normally. There were clear signs of arthritis at both ankles (Figures 1 and 2) and at the left wrist. The referred joints were tender and swollen at the physical exam. There were also discrete petechiae (Figures 1 and 2).

Prednisone 20 mg was started after a few days with improvement in articular signs. Laboratorial exams showed a high white cell count, 15,9 × 10^9^ (reference: 4–10 × 10^9^) with neutrophilia, elevated C-reactive protein (CRP), 41,5 mg/dL (reference: < 1 mg/dL), and hemosedimentation rate of 120 mm/h (reference: < 15 mm). Arthrocentesis was not performed due to polyarticular involvement. Three days after her admission two hemocultures showed* Neisseria meningitidis* serogroup C. After these results a cerebrospinal fluid analysis was performed and showed no significant alterations. The patient did not present fever, neurological dysfunction, or hemodynamic alteration at the follow-up. Intravenous ceftriaxone 2 g twice daily was started and prednisone increased to 60 mg. Seven days after the beginning of the treatment, she was asymptomatic, with CRP of 2,4 mg/dL, and was discharged home. All patient close contacts were prescribed ciprofloxacin as a chemoprophylaxis.

## 3. Discussion

Gram-negative diplococci* Neisseria meningitidis* is present between 5 and 10% at the upper airways of healthy adults [1]. Its infection in predisposing subjects begins through the contact with contaminated airdrops into the pharynx, followed by blood dissemination [5]. Smoking, viral upper airway infection, splenectomy or functional asplenia, and complement impairment are the main risk factor to this type of infection [6].

*Neisseria meningitidis* serogroup B is the most common group in numerous countries, mainly in Europe and South America. There is an association between low socioeconomic levels and this disease, but the main cause of the difference in prevalence is the underreporting of cases. Routine serogroup C vaccination programs have played an important role in reducing carriage rates and the incidence of disease as well as inducing herd immunity [7].

Primary septic arthritis is predominantly due to Gram-positive cocci (*Staphylococcus sp.* and* Streptococcus sp.*)*. Neisseria meningitidis* is the etiologic agent around 1% of reported cases [6].

Joint involvement is common secondary to meningitis and disseminated meningococcal disease, reaching up to 4 to 50% of subjects [8, 9]. The primary meningococcal arthritis is a rare manifestation of the infection. Until 2002 there were 34 reported cases of primary meningococcal arthritis [9]. Joint involvement has a variable pattern with the knees being the most common joint affected. Polyarticular involvement occurs in around 30% of the cases. At 50% of the cases reported an upper airway infection preceded the arthritis [6].

The patient described here presented a type C* Neisseria meningitidis.* A recent study suggested an association between the serogroup C and polyarticular manifestation [10]. Among the primary meningococcal arthritis the serogroup C was associated in 36%, group B in 30%, and group W-135 in 13% [11].

Diagnosis is suspected based on the history of recent sore throat, fever, and mono- or polyarthritis of large joints associated with skin lesions. Leukocytosis with neutrophil predominance and high reactive protein C and hemosedimentation rate are common findings. Culture of the synovial fluid becomes positive in 90% of the cases and blood cultures can reach 40% of positivity [6].

Arthritis with meningococcal disease frequently manifests as an immune-mediated form with sterile effusions affecting large joints, often with polyarthritis and fever. Arthritis onset is between 1 and 12 days after the initial illness [12].

A broad-spectrum antibiotic such as penicillin or third generation cephalosporin for a short-term (around 7 days) can be successful in the majority of cases possibly due to a lack of synovial or cartilage involvement. There is no consensus about the use of corticosteroids. However, some studies suggested corticosteroids prescription because of immune-mediated reactions associated with the disease [8, 9, 13].

Joint prognosis is generally good [5]. A recent Spanish study showed that no patient with primary meningococcal arthritis evolved to joint deformation or impairment at long-term follow-up [13].

One limitation of this case report is the absence of synovial fluid analyses, which could prove a true infection in the synovial fluid with bacterial isolation or a purulent synovial fluid, although we believe that the isolation of* Neisseria meningitidis* serogroup C in 2 hemocultures in a patient with arthritis makes primary meningococcal polyarthritis the main diagnostic.

Despite of its rarity the primary meningococcal arthritis must be considered a differential diagnosis among early onset arthritis preceded by upper airway infection due to its potential good prognosis when properly treated.
